# 722 Factors Affecting Split Thickness Skin Graft Loss in Elderly Burn Patients

**DOI:** 10.1093/jbcr/irae036.265

**Published:** 2024-04-17

**Authors:** Matthew J Reiss, Naiwei Hsu Chang

**Affiliations:** Torrance Memorial Medical Center, Torrance, California; Torrance Memorial Medical Center, Torrance, California

## Abstract

**Introduction:**

Advanced age is one of the more established predictors of morbidity and mortality in patients with burn injuries. It is predicted that by 2030, the number of people 65 years or older may double. This demographic shift may lead to a concomitant increase in burn injuries. The elderly population is at higher risk for burn injuries due to physiological changes that includes impairment of cognition, coordination, vision, mobility and deterioration of reflexes which may lead to decreased responses in dangerous situations. Other factors that may impair the healing process can include increased co-morbidities, polypharmacy, malnutrition in the setting of hypermetabolism, and decreased physiological reserves. Due to this, there is an increased incidence of graft loss in the elderly burn population. Our study aims to identify other factors that may affect or contribute to graft loss in elderly burn patients.

**Methods:**

A retrospective chart review was performed on patients aged 65 years old and above who were admitted at our Burn Center for Burn injuries from January 2020 to September 2022. Medical Records of 54 patients were examined with regards to patients’ demographics, co-morbidities, site of wound graft placement, and technique used in wound graft application. Graft loss, which was determined to be < 80% take, was examined at 1-week intervals in the post-operative period and patient outcomes at 1 to 2 weeks were included.

**Results:**

It was noted that out of the 54 patient charts reviewed, 24 patients underwent Split Thickness Skin Graft with autologous skin donor. The population consisted of more male (70.8%) than female (29.2%) patients. The average graft take 1 week after surgery was 87.5% and 80.2% at 2 weeks after surgery. About 33.3% of this geriatric burn patients suffered graft loss. Half of these patients were noted to have Diabetes Mellitus, Hypertension and/or had a significant smoking history. The most frequently used surgical grafting technique was meshed skin grafts. It was observed that 50% of patients who sustained graft loss had 4:1 meshed graft placement and 37.5% had 2:1 meshed skin graft placement. 40% of the skin graft loss were on the upper extremities and 37.5% were on lower extremities.

**Conclusions:**

Elderly burn patients in our population who have DM, HTN, smoking history have a higher incidence of graft loss. These patients have improved outcome with 2:1 meshed autograft and the location of the graft placement did not play a significant difference in graft take.

**Applicability of Research to Practice:**

The aim of this retrospective study is to indicate and predict populations with increased risk factors to formulate a therapeutic plan of action to decrease possibility of graft failure. More effective methods may be implemented to avoid increased incidence of graft loss such as use of bolster dressing vs NPWT, orthopedic devices such as splints, and close monitoring of skin graft using bedside measurement tools.

